# Influence of Adult Height on Rheumatoid Arthritis: Association with Disease Activity, Impairment of Joint Function and Overall Disability

**DOI:** 10.1371/journal.pone.0064862

**Published:** 2013-05-21

**Authors:** Ying Chen, Zanzhe Yu, Jonathan C. Packham, Derek L. Mattey

**Affiliations:** 1 Haywood Rheumatology Centre, Haywood Hospital, High Lane, Burslem, Stoke-on-Trent, Staffordshire, United Kingdom; 2 Institute of Science and Technology in Medicine, Keele University, Staffordshire, United Kingdom; University of Michigan Medical School, United States of America

## Abstract

**Objectives:**

To investigate whether normal variation of adult height is associated with clinical characteristics in rheumatoid arthritis (RA), including disease activity (DAS28), impairment of joint function (mechanical joint score, MJS) and overall disability (health assessment questionnaire, HAQ).

**Methods:**

A cohort (134 males, 287 females) of consecutively recruited RA patients of Northern European origin was studied. Height, weight and demographic information were obtained. A core set of disease measurements, including DAS28, MJS and HAQ, were recorded at baseline, 12 and 24 months. Other clinical variables (e.g. disease duration, IgM rheumatoid factor, antibodies to cyclic citrullinated peptide, C-reactive protein, erythrocyte sedimentation rate) were recorded at baseline. Socioeconomic status, smoking status, comorbid condition, other autoimmune conditions and drug therapy were also recorded. Associations were analyzed using univariate statistics and multivariate linear regression models. Mediation tests were also carried out for evaluating the relationship between gender, height and disease measures.

**Results:**

In males, height was inversely associated with DAS28, MJS and HAQ (at baseline and over 24 months) independent of other factors (e.g. weight, body mass index, age, disease duration, osteoporosis, autoantibodies, erosive disease, joint replacement, steroid use, smoking status, socioeconomic status and comorbid disease). In females, a similar trend was seen but the relationships were non significant. In the whole population, the association of female gender with more active disease and poor function disappeared after adjustment for height. Mediation analysis indicated that height served as a full mediator in the relationship of gender with disease activity and overall disability. Confirmation of these findings was demonstrated in a second RA population (n = 288).

**Conclusion:**

Adult height is inversely associated with disease activity, impairment of joint function and overall disability in RA, particularly in males. The association of female sex with more severe disease activity and disability appears to be mediated by smaller stature.

## Introduction

Body height is among the most visible of human characteristics, and is highly heritable (h^2^ = 0.8) [1]. It has been associated with numerous genomic loci (n>100), with each contributing a small amount of effect [2]. It is a complex trait also influenced by a variety of environmental factors, including diet and the prenatal environment [3].

Normal variation of height in adulthood is associated with several disease conditions, including various cancers (short stature/decreased risk) [4], [5], cardiovascular diseases (CVD) (short stature/increased risk) [6], type 2 diabetes (short stature/increased risk) [7], periodontitis (short stature/increased risk) [8], and chronic obstructive pulmonary disease (short stature/increased risk) [9]. Previous studies have found no relationship between height and the risk of developing rheumatoid arthritis (RA) [10], [11], but as far as we are aware there have been no studies on whether there is a relationship between height and disease activity or severity in rheumatoid arthritis (RA).

It has been suggested that the association of short stature with CVD and other diseases may be related to an increased inflammatory burden in such individuals due to early-life infections which have impact on eventual adult height [12]. We hypothesized that there may be an association between adult height and disease activity and/or severity in patients with RA. In the present study we investigated whether there was a relationship between height and a number of major disease characteristics in RA, including disease activity, impairment of mechanical joint function and global degree of disability. Our results suggest that, in men particularly, height is inversely associated with increased disease activity, and overall severity in RA. The well described association of female sex with more severe disease activity and poor functional outcome appears to be mediated by smaller stature.

## Methods

### Patients

This study was based on a cohort (n = 430) of consecutively recruited RA patients of Northern European origin, resident in North Staffordshire and attending the Clinical Rheumatology Unit at the Haywood Hospital. All patients had a diagnosis of RA, and met the 1987 American College of Rheumatology criteria [13]. Nine (2.1%) samples were excluded from the current report, on the basis that information on height, sex or any key outcome variable was incomplete. Inclusion or exclusion of these samples made no difference to the associations found.

Body height (standing height) and weight were measured on each patient at baseline. Height (in cm to the nearest 0.1 cm) was measured with a stadiometer with a measuring slide and a heel plate. Position of the head was standardized by asking the sampled subject to stand straight, without shoes and with the heels together. Weight (in kg to the nearest 0.1 kg) was measured with a reliable weighing scale while the participant was wearing light clothing and no shoes. Body mass index (BMI) was obtained by weight (in kg)/height^2^ (in m). Five of the 421 patients were wheelchair users, but with support it was possible for them to stand straight enough for height measurements. All of these patients were women. Other demographic data (e.g. age, gender, post code of residence, occupation) was also recorded at recruitment. Socioeconomic status was estimated by the Carstairs index of deprivation based on postcode address [14]. A history of current or past cigarette smoking was obtained from a questionnaire completed by each patient at baseline, as described previously [15].

Clinical variables, including disease duration, disease activity, IgM rheumatoid factor (RF), anti-cyclic citrullinated peptide antibody (anti-CCP), levels of C-reactive protein (CRP) and erythrocyte sedimentation rate (ESR), presence of extra-articular disease (e.g. nodules, vasculitis, interstitial lung disease), and presence of erosive disease were obtained at baseline. RA disease activity was measured by the Disease Activity Score 28 (DAS28). Evaluation of DAS28 includes the number of swollen and tender joints (based on 28 joints), the global visual analogue scale, and ESR [16]. The Mechanical Joint Score (MJS) and the Health Assessment Questionnaire (HAQ) score were used to assess the total amount of joint impairment of mechanical function and the overall degree of disability, respectively [17], [18]. These three outcome variables were obtained at baseline, 12 and 24 months follow-up. Complete data at all time points over 24 months was available on 305/430 (72.4%) patients.

Patients were also categorized into those with or without osteoporosis at the time of study entry. Patients were classified has having osteoporosis if their T scores obtained by bone densitometry were less than −2.5, and/or if they were being treated with anti-osteoporotic drugs. Previous/current medication and any major comorbid conditions (e.g. ischaemic heart disease (IHD), diabetes, chronic pulmonary disease, renal disease, neoplasia) on each patient were recorded at recruitment, as described previously [15]. Other variables recorded included evidence of other autoimmune conditions (e.g. Sjogren's syndrome, autoimmune thyroid disease, inflammatory bowel disease), any hypothyroidism, and any history of knee or hip joint replacement.

A second study population of patients (n = 288, median age 62.0, median disease duration 8.0 years, female n = 175) was used in a replication study of the association of height with clinical characteristics. DAS28 scores were obtained on the majority of patients (n = 285), although HAQ scores were only available for 94 patients. MJS data were not collected. This group was on standard disease modifying anti-rheumatic drug (DMARD) therapy and had been recruited as a control group for patients on biologic therapy in previous studies.

### Ethics statement

Written informed consent was provided by each patient according to the Declaration of Helsinki. The research was approved by the North Staffordshire Local Research Ethics Committee.

### Statistical analysis

The relationship between binary variables was assessed using contingency tables. The relationship between binary variables and quantitative variable was assessed using two-sample T-test (Student's, Aspin-Welch or Mann-Whitney U Test, according to the distribution and the variance). Correlation testing (Pearson's or Spearman's Correlation, according to the distribution) was used to assess the relationship between quantitative variables. Multivariate linear modelling (multivariate multiple regression) was used to assess the influence of independent variable (e.g. body height, sex) on dependent variable (e.g. disease activity) with or without adjustment for other covariates. The selection of other covariates in each model was based on an all possible regressions procedure to determine which candidate variables should be included in the final model. This algorithm seeks a subset that provides a maximum value of R-squared. Multiple regression analyses were then run to determine the regression coefficients and p values for each of the independent variables chosen in the final model. Each regression analysis was examined for multicollinearity by looking at the variance inflation factors, tolerances and condition number test. The mediation effect was assessed by using the Preacher and Hayes' method for assessing and comparing indirect effects in mediator models [19]. The macro for this method, called INDIRECT, can be accessed at http://www.afhayes.com/spss-sas-and-mplus-macros-and-code.html. This macro estimates the path coefficients and generates bootstrap confidence intervals for indirect effects through mediator variables, and allows for adjustment for other covariates. Analyses were all carried out using the Number Cruncher Statistical System for Windows (version NCSS 2007), except for the mediation tests (SPSS version 20 with the INDIRECT macro). The significance level was set at a p value of 0.05, and all p values were based on 2-tailed tests. MJS did not fit a normal distribution and was square root transformed in multiple regression models to fit normality.

## Results

### Characteristics of patients

The cohort consisted of 134 (31.8%) male and 287 female patients with RA. Table 1 shows the demographic and clinical variables at baseline. In this particular population, female patients were younger, and a significantly higher percentage of female patients were currently receiving methotrexate. As expected, females were shorter with lower body weight. There was no difference in BMI between genders. Ever-smoking was less frequent in females than males. Female patients with RA were associated with earlier onset, higher DAS28 (non-significant), higher HAQ score, and a greater frequency of osteoporosis and hypothyroidism. Males were more likely to be associated with common co-morbid conditions (e.g. hypertension, IHD, diabetes). These remained significantly associated after adjustment for age and disease duration.

**Table 1:** Demographic and clinical characteristics compared between female and male patients.

Parameter	All patients, n = 421	Male, n = 134	Female, n = 287	p value¶
Demographic variable				
Age, yrs	62.0 (54.0–69.0)	64.5 (57.8–72.0)	60.0 (54.0–67.0)	0.0002
Body weight, kg	76.2±15.3	84.2±13.6	72.4±14.6	<0.0001
Height, cm	165.3±9.0	174.2±6.5	161.1±6.6	<0.0001
BMI, kg/m^2^	27.8±5.1	27.8±4.4	27.9±5.5	>0.10
Ever-smoking	275/418 (65.8)	109/133 (82.0)	166/285 (58.2)	<0.0001
Carstairs score	1.10 (−1.63–3.0)	1.30 (−2.55–3.25)	1.10 (−1.50–3.0)	>0.10
RA clinical variable				
Age at onset, yrs	49.6±13.1	51.8±12.6	48.5±13.2	0.016
Duration, yrs	9.0 (3.0–17.3)	8.5 (4.0–18.3)	9.0 (2.7–17.0)	>0.10
RF status	238/419 (56.8)	75/133 (56.4)	163/286 (57.0)	>0.10
Anti-CCP status	308/408 (75.5)	96/130 (73.8)	212/278 (76.3)	>0.10
CRP, mg/l	11.0 (4.5–20.0)	11.8 (5.0–22.9)	10.3 (4.0–19.0)	>0.10
ESR, mm/hrs†	20.0 (10.0–35.8)	16.0 (7.5–37.0)	22.0 (11.0–35.0)	0.067
PGA†	43.0 (21.0–57.0)	46.0 (22.0–62.5)	39.5 (20.8–54.0)	>0.10
TJC†	4 (1–9)	3 (1–8)	4 (1–9)	>0.10
SJC†	3 (1–6)	2 (0–5)	3 (1–7)	>0.10
DAS28	4.20±1.38	4.05±1.48	4.27±1.33	>0.10
MJS	7.0 (3.0–15.0)	7.0 (3.0–14.0)	7.0 (3.0–16.0)	>0.10
HAQ score	1.625 (1.0–2.0)	1.50 (0.875–2.0)	1.625 (1.125–2.125)	0.046
Osteoporosis	59/421 (14.0)	12/134 (9.0)	47/287 (16.4)	0.041
Joint replacement*	66/419 (15.8)	24/133 (18.0)	42/286 (14.7)	>0.10
Drug treatment for RA				
DMARD use	392/421 (93.1)	122/134 (91.0)	270/287 (94.1)	>0.10
Methotrexate use	255/421 (60.6)	71/134 (53.0)	184/287 (64.1)	0.030
Steroid use	41/420 (9.8)	15/134 (11.2)	26/286 (9.1)	>0.10
Biologic agent use	60/421 (14.3)	19/134 (14.2)	41/287 (14.3)	>0.10
Co-morbid condition				
Hypertension	166/418 (39.7)	66/134 (49.3)	100/284 (35.2)	0.0062
IHD	84/420 (20.0)	43/133 (32.3)	41/287 (14.3)	<0.0001
Diabetes‡	32/420 (7.6)	17/133 (12.8)	15/287 (5.2)	0.0066
Hypothyroidism	27/420 (6.4)	1/133 (0.75)	26/287 (9.05)	0.001
Asthma	44/419 (10.5)	13/133 (9.8)	31/286 (10.8)	>0.10

Values are n (%), mean ± standard deviation (SD) or median (interquartile range (IQR)).

¶ Comparison between male and female patients, un-adjusted;

* hip or knee replacement;

† components of DAS28;

‡ types I and II combined.

### Body height and RA in sex-separated analysis

Body height in males ranged between 157 and 197 cm, and in females between 146 and 180.5 cm. Table 2 shows the selected characteristics compared between patients below (<), and equal or above (≥) median height for male (173.3 cm) and female patients (160.5 cm) separately.

**Table 2:** Demographic and clinical characteristics compared between patients with below (<) and equal or above (≥) median height in sex-separated setting.

Parameter	Male patients, n = 134			Female patients, n = 287		
	<median, n = 67	≥median, n = 67	p value¶	<median, n = 143	≥median, n = 144	p value¶
Age, yrs	65.0 (59.0–72.0)	64.0 (52.0–72.0)	>0.10	62.0 (54.0–69.0)	59.0 (53.3–66.0)	>0.10
Body weight, kg	80.8±12.7	87.6±13.7	0.0034	68.8±13.5	75.9±14.8	<0.0001
BMI, kg/m^2^	28.3±4.6	27.3±4.2	>0.10	28.3±5.5	27.4±5.4	>0.10
Ever smoke	53/67 (79.1)	56/66 (84.8)	>0.10	85/142 (59.9)	81/143 (56.6)	>0.10
Carstairs score	1.30 (−1.40–3.40)	1.15 (−2.73–2.88)	>0.10	1.20 (−1.60–3.10)	1.10 (−1.40–3.0)	>0.10
Duration, yrs	7.0 (4.0–14.0)	11.0 (5.0–21.0)	0.019	11.0 (3.0–18.0)	9.0 (2.50–17.0)	>0.10
RF status	36/66 (54.5)	39/67 (58.2)	>0.10	85/143 (59.4)	78/143 (54.5)	>0.10
Anti-CCP status	48/65 (73.8)	48/75 (73.8)	>0.10	112/138 (81.2)	100/140 (71.4)	0.057
CRP, mg/l	12.3 (5.0–31.2)	11.0 (4.8–20.0)	>0.10	10.0 (4.3–19.0)	11.0 (4.0–19.0)	>0.10
ESR, mm/hrs†	16.0 (9.0–44.3)	15.0 (6.0–28.0)	0.071	22.0 (12.0–38.0)	19.5 (11.0–34.0)	>0.10
PGA†	46.0 (33.0–60.0)	46.0 (16.8–65.5)	>0.10	43.0 (23.0–54.0)	36.0 (19.0–54.0)	>0.10
TJC†	3 (2–8)	3 (0–7)	>0.10	5 (1–10)	4 (1–8)	>0.10
SJC†	2 (1–6)	2 (0–5)	>0.10	3 (1–6)	3 (1–7)	>0.10
DAS28	4.34±1.31	3.75±1.59	0.023	4.34±1.37	4.20±1.29	>0.10
MJS	9.0 (3.0–17.0)	6.0 (2.8–12.0)	0.076	8.0 (3.0–17.0)	6.0 (3.0–15.0)	>0.10
HAQ score	1.75 (1.25–2.125)	1.25 (0.625–1.875)	0.0046	1.75 (1.25–2.125)	1.625 (1.0–2.0)	>0.10
Osteoporosis	10/67 (14.9)	2/67 (3.0)	0.016	28/143 (19.6)	19/143 (13.2)	>0.10
Joint replacement*	13/67 (19.4)	11/66 (16.7)	>0.10	20/142 (14.1)	22/142 (15.3)	>0.10
DMARD use	60/67 (89.6)	62/67 (92.5)	>0.10	136/143 (95.1)	134/144 (93.1)	>0.10
Methotrexate use	38/67 (56.7)	33/67 (49.3)	>0.10	86/143 (60.1)	98/144 (68.1)	>0.10
Steroid use	6/67 (9.0)	9/67 (13.4)	>0.10	14/143 (9.8)	12/143 (8.4)	>0.10
Biologic agent use	12/67 (17.9)	7/67 (10.4)	>0.10	20/143 (14.0)	21/144 (14.6)	>0.10
Hypertension	34/67 (50.7)	32/67 (47.8)	>0.10	57/141 (40.4)	43/143 (30.1)	0.068
IHD	20/67 (29.9)	23/66 (34.8)	>0.10	25/143 (17.5)	16/144 (11.1)	>0.10
Diabetes‡	7/67 (10.4)	10/66 (15.2)	>0.10	10/143 (7.0)	5/144 (3.5)	>0.10
Hypothyroidism	1/67 (1.5)	0/66 (0.0)	>0.10	13/143 (9.1)	13/144 (9.0)	>0.10
Asthma	7/67 (10.4)	6/66 (9.1)	>0.10	21/142 (14.8)	10/144 (6.9)	0.033

Values are n (%), mean ± SD or median (IQR).

¶ Comparison within gender, un-adjusted;

* hip or knee replacement;

† components of DAS28;

‡ types I and II combined.

It was a prerequisite of the present study to avoid the confounding effect of age on body height and on other parameters e.g. the younger generation tends to be taller, and increasing age may be associated with reduction in height. The correlations between height (continuous variable) and age in males and females were (r = −0.22, p = 0.010) and (r = −0.15, p = 0.014), respectively. All analyses were therefore adjusted for age.

### Association with disease activity and outcome indices

In males, patients <median height were associated with higher DAS28, MJS (borderline association) and HAQ score, compared to those ≥median height (Table 2). The correlations of height (continuous variable) with DAS28, MJS and HAQ score were (r = −0.25, p = 0.0039), (r = −0.22, p = 0.0095) and (r = −0.23, p = 0.0066), respectively. In females, the association of <median height with worse RA outcome (DAS28, MJS and HAQ score) was not significant, but showed a similar trend to that in males (Table 2). Correlation analysis indicated that height (continuous variable) was significantly correlated with HAQ score (r = −0.14, p = 0.017) but not with DAS28 or MJS.

To determine whether demographic, clinical and other variables were likely to be potential confounders or effect modifiers of the associations with height we carried out univariate linear regression analyses to determine which variables were most significantly associated with disease activity/severity measures. Variables with a p value of 0.2 or less were chosen for inclusion in further multivariate analyses. An all possible regressions procedure was run for each disease measure to determine which subset of candidate variables should be run in the final multiple regression models. Introduction of weight, BMI or other variables into these multivariate models made little or no difference to the association with height in men, although a number of other variables were also significant in these models. Notably, the presence of any co-morbid disease was significantly associated with DAS28 and HAQ, while the use of steroids was independently associated with HAQ and MJS. The presence of RF was associated only with DAS28, CRP was associated only with MJS, and the Carstairs index was associated only with the HAQ. (Table 3).

**Table 3:** Multivariate multiple regression analyses showing variables most strongly associated with DAS28, MJS and HAQ score in male patients with RA.

Model 1, dependent variable: DAS28			Model 2, dependent variable: MJS*			Model 3, dependent variable: HAQ		
Independent variable	Regression coefficient (SE)	p value	Independent variable	Regression coefficient (SE)	p value	Independent variable	Regression coefficient (SE)	p value
Height, cm	−0.050 (0.018)	0.005	Height, cm	−0.058 (0.016)	0.0008	Height, cm	−0.026 (0.009)	0.006
Comorbid disease†	1.682 (0.412)	<0.0001	Duration, yrs	0.074 (0.010)	<0.0001	Duration, yrs	0.018 (0.0060)	0.0027
RF (+/−)	0.572 (0.239)	0.018	CRP mg/l	0.009 (0.004)	0.015	Comorbid disease†	0.771 (0.207)	0.0003
			Steroid use	0.870 (0.346)	0.013	Carstairs index	0.064 (0.021)	0.003
						Steroid use	0.454 (0.190)	0.018

Variables were baseline values.

* MJS was square root transformed to fit normality.

† Presence of any comorbid disease (e.g ischaemic heart disease, diabetes, chronic pulmonary disease, renal disease, neoplasia).

RF, rheumatoid factor; CRP, C-reactive protein.

R-squared values: Model 1 = 0.2320, Model 2 = 0.3967, Model 3 = 0.2760.

In females, height was not significantly associated with any of the disease activity/severity parameters in multivariate models which included other significant variables (Table 4). Other anthropometric measurements (weight or BMI) were not significantly associated. As in men, co-morbid disease and presence of RF was associated with DAS28, although steroid use was also associated in women. The strongest associations with MJS were disease duration, presence of RF and osteoporosis, while the HAQ was associated with disease duration, ESR, Carstairs index, steroid use and hip replacement.

**Table 4:** Multivariate multiple regression analyses showing variables most strongly associated with DAS28, MJS and HAQ score in female patients with RA.

Model 1, dependent variable: DAS28			Model 2, dependent variable: MJS*			Model 3, dependent variable: HAQ		
Independent variable	Regression coefficient (SE)	p value	Independent variable	Regression coefficient (SE)	p value	Independent variable	Regression coefficient (SE)	p value
RF (+/−)	0.607 (0.153)	<0.0001	Duration, yrs	0.098 (0.0089)	<0.0001	Duration, yrs	0.025 (0.0044)	<0.0001
Comorbid disease†	0.594 (0.211)	0.005	RF (+/−)	0.618 (0.150)	<0.0001	ESR, mm/h	0.014 (0.002)	<0.0001
Steroid use	0.534 (0.262)	0.042	Osteoporosis	0.879 (0.202)	<0.0001	Carstairs index	0.049 (0.016)	0.003
						Steroid use	0.329 (0.137)	0.017
						Hip replacement	0.296 (0.140)	0.035

Variables were baseline values.

* MJS was square root transformed to fit normality.

† Presence of any comorbid disease (e.g ischaemic heart disease, diabetes, chronic pulmonary disease, renal disease, neoplasia).

RF, rheumatoid factor; ESR, erythrocyte sedimentation rate.

R-squared values: Model 1 = 0.1023, Model 2 = 0.4189, Model 3 = 0.2721.

The relationships between height and the mean-time-averaged DAS28, MJS and HAQ score over 24 months (MTA-DAS28, MJS, and HAQ score at baseline, 12 and 24 months) were also analyzed on 103 (76.9%) male and 202 (70.4%) female patients using similar regression models. Height showed very significant associations with MTA-DAS28, MTA-MJS and MTA-HAQ score in males after adjusting for other significant independent variables (Table 5). In females there were no associations between height and mean time-averaged disease measures in models containing the most significant baseline variables associated with each measure (Table 6). BMI was significantly associated with the MTA-DAS28 in a model which also included RF and comorbid disease.

**Table 5:** Multivariate multiple regression analyses showing baseline variables most strongly associated with DAS28, MJS and HAQ score over time (24 months) in male patients with RA.

Model 1, dependent variable: MTA-DAS28			Model 2, dependent variable: MTA-MJS*			Model 3, dependent variable: MTA-HAQ		
Independent variable	Regression coefficient (SE)	p value	Independent variable	Regression coefficient (SE)	p value	Independent variable	Regression coefficient (SE)	p value
Height, cm	−0.063 (0.018)	0.0005	Height, cm	−0.055 (0.017)	0.0018	Height, cm	−0.031 (0.009)	0.0016
Comorbid disease†	1.470 (0.434)	0.001	Duration, yrs	0.069 (0.011)	<0.0001	Comorbid disease†	0.717 (0.234)	0.003
			Steroid use	1.090 (0.323)	0.0011	Duration, yrs	0.017 (0.0068)	0.005
						Carstairs index	0.056 (0.022)	0.011
						Steroid use	0.414 (0.201)	0.042

MTA: mean-time-averaged values over 24 months, scored at baseline, 12 and 24 months. Based on 103 (76.9%) male patients who had been followed up for 24 months with measurements at each time point available. Independent variables were baseline values.

* MTA-MJS was square root transformed to fit normality. ,

† Presence of any comorbid disease (e.g ischaemic heart disease, diabetes, chronic pulmonary disease, renal disease, neoplasia).

R-squared values: Model 1 = 0.2327, Model 2 = 0.3967, Model 3 = 0.3058.

**Table 6:** Multivariate multiple regression analyses showing baseline variables most strongly associated with DAS28, MJS and HAQ score over time (24 months) in female patients with RA.

Model 1, dependent variable: MTA-DAS28			Model 2, dependent variable: MTA-MJS*			Model 3, dependent variable: MTA-HAQ		
Independent variable	Regression coefficient (SE)	p value	Independent variable	Regression coefficient (SE)	p value	Independent variable	Regression coefficient (SE)	p value
RF (+/−)	0.018 (0.007)	0.0036	Duration, yrs	0.093 (0.0086)	<0.0001	Duration, yrs	0.025 (0.0047)	<0.0001
BMI	0.038 (0.014)	0.0088	RF (+/−)	0.435 (0.160)	0.007	ESR, mm/h	0.009 (0.002)	0.0002
Age, yrs	0.018 (0.0076)	0.020				Carstairs index	0.045 (0.018)	0.012
Comorbid disease†	0.437 (0.215)	0.044				Hip replacement	0.350 (0.159)	0.033

MTA: mean-time-averaged values over 24 months, scored at baseline, 12 and 24 months. Based on 202 (70.4%) female patients who had been followed up for 24 months with measurements at each time point available. Independent variables were baseline values.

* MTA-MJS was square root transformed to fit normality.

† Presence of any comorbid disease (e.g ischaemic heart disease, diabetes, chronic pulmonary disease, renal disease, neoplasia).

RF, rheumatoid factor; BMI, body mass index; ESR, erythrocyte sedimentation rate.

R-squared values: Model 1 = 0.1464, Model 2 = 0.4056, Model 3 = 0.2358.

### Gender vs. height in association with disease activity and outcome indices

Comparison between genders showed that female patients tended to have higher scores for the DAS28 and HAQ score, although the DAS28 association was not significant in univariate analysis (Table 1). We hypothesized that the association of females with more severe RA may be related to females having a generally shorter stature than males.

In multivariate multiple regression models of the whole population, female sex was associated with higher DAS28 and HAQ scores after adjusting for other variables, but excluding height (Table S1). However, after inclusion of height (continuous variable) as a covariate in these models, the association of female sex with higher DAS28 and HAQ score totally disappeared (Table 7). The association of height with disease activity/severity measures was significant in all models with or without adjustment for gender, although the significance levels for the DAS28 and HAQ associations were attenuated after adjusting for gender (Table 7). Height also showed significant associations with MTA-DAS28, MTA-MJS and MTA-HAQ score over 24 months after adjusting for other confounding factors (Table S2).

**Table 7:** Multivariate multiple regression analyses showing variables most strongly associated with DAS28, MJS and HAQ score in all patients with RA (adjusted for gender).

Model 1, dependent variable: DAS28			Model 2, dependent variable: MJS*			Model 3, dependent variable: HAQ		
Independent variable	Regression coefficient (SE)	p value	Independent variable	Regression coefficient (SE)	p value	Independent variable	Regression coefficient (SE)	p value
Height, cm	−0.0222 (0.009)	0.023	Height, cm	−0.0142 (0.007)	0.029	Height, cm	−0.0122 (0.003)	0.019
RF (+/−)	0.605 (0.130)	<0.0001	Duration, yrs	0.085 (0.006)	<0.0001	Duration, yrs	0.021 (0.003)	<0.0001
Comorbid disease†	0.841 (0.190)	<0.0001	Osteoporosis	0.769 (0.183)	<0.0001	ESR, mm/h	0.008 (0.002)	<0.0001
Steroid use	0.500 (0.216)	0.021	CRP, mg/l	0.0084 (0.0026)	0.002	Carstairs index	0.059 (0.013)	<0.0001
Female	−0.0145 (0.189)	0.93	RF (+/−)	0.295 (0.126)	0.020	Comorbid disease†	0.318 (0.098)	0.001
			Female	−0.190 (0.182)	0.30	Steroid use	0.308 (0.114)	0.007
						Hip replacement	0.234 (0.117)	0.048
						Female	0.048 (0.098)	0.62

Variables were baseline values.

* MJS was square root transformed to fit normality,

† Presence of any comorbid disease (e.g ischaemic heart disease, diabetes, chronic pulmonary disease, renal disease, neoplasia).

RF, rheumatoid factor; CRP, C-reactive protein; ESR, erythrocyte sedimentation rate. Models shown adjusted for gender. In models unadjusted for gender the associations with height remained significant (DAS28, p = 0.002; MJS, p = 0.043, HAQ, p = 0.0002).

R-squared values: Model 1 = 0.1359, Model 2 = 0.3833, Model 3 = 0.2792.

**Figure 1:**
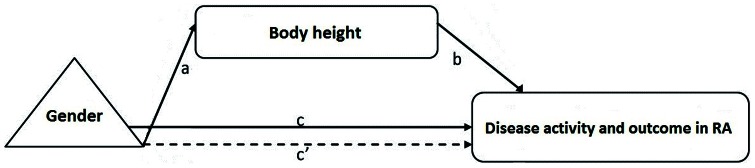
Mediation model for height as a mediator in the relationship between gender and disease measures in RA. Gender, independent variable; body height, mediator variable; disease activity and outcome in RA, dependent variable. Path of effect: a, effect of independent variable on mediator variable; b, effect of mediator variable on dependent variable; c, total effect of independent variable on dependent variable; a×b, indirect effect of independent variable on dependent variable; c', direct effect of independent variable on dependent variable; c = c'+a×b.

We postulated that height may be acting as a mediator in the relationship of gender, with disease activity and outcome indices (Figure 1). To formally investigate this, we applied Preacher and Hayes' method for assessing and comparing indirect effects in mediator models using the INDIRECT macro. The results demonstrated that although the total effect of gender on DAS28 and HAQ was significant (c path, models 1 and 3 respectively, Table 8), the direct effect contributed little or nothing to this (c' path, models 1 and 3 respectively, Table 8). The indirect effect through the mediator (i.e. height) played the major role, based on the significance of both a and b paths, and the quantified a×b effect (a, b, a×b paths, models 1 and 3 respectively, Table 8). Gender did not have a significantly effect on MJS (c path, model 2, Table 8). However, the effect of height on MJS was significant (b path, model 2, Table 8). The analyses were adjusted for age and disease duration, and further adjustment made no significant difference to the results (data not shown). Similar results were also obtained on index records over 24 months (Table S3).

**Table 8:** Mediation models demonstrating the mediator effect of height on the relationship between gender and measures of disease activity and outcome in all patients with RA.

Model 1, dependent variable: DAS28				Model 2, dependent variable: MJS				Model 3, dependent variable: HAQ			
Path of effect	Regression coefficient (SE)	p value	*Bootstrap results: regression coefficient (95% CI)	Path of effect	Regression coefficient (SE)	p value	*Bootstrap results: regression coefficient (95% CI)	Path of effect	Regression coefficient (SE)	p value	*Bootstrap results: regression coefficient (95% CI)
a	−13.491 (0.690)	<0.0001	-	a	−13.491 (0.690)	<0.0001	-	a	−13.501 (0.688)	<0.0001	-
b	−0.0226 (0.010)	0.031	-	b	−0.0285 (0.098)	0.0037	-	b	−0.0177 (0.005)	0.0009	-
a×b	0.305 (-)	-	0.301 (0.019–0.579)	a×b	0.384 (-)	-	0.383 (0.113–0.671)	a×b	0.239 (-)	-	0.239 (0.093–0.380)
c	0.298 (0.146)	0.043	-	c	0.156 (0.138)	0.26	-	c	0.232 (0.075)	0.0021	-
c'	−0.0070 (0.203)	0.97	-	c'	−0.229 (0.190)	0.23	-	c'	−0.0074 (0.103)	0.94	-

Gender as the independent variable; height as the proposed mediator; disease activity and outcome index (i.e. DAS28, MJS or HAQ) at baseline as dependent variable. MJS was square root transformed to fit normality. Models shown adjusted for age and disease duration as standard; adjustment for further confounding factors made no significant difference to the results. Path of effect: a, effect of gender on height; b, effect of height on disease indices; a×b, indirect effect through mediation; c, total effect; c', direct effect; c can be expressed as the sum of the direct and indirect effects (i.e. c = c'+a×b).

* Bootstrap results for indirect effect through proposed mediation: number of bootstrap resamples, n = 5,000.

To investigate whether sex influenced disease measurements in patients with equal height, we examined a subgroup of 54 females with height between 161.4 and 180.5 cm and a randomly selected group of 54 height matched males with height between 161.4 and 180.5 cm. This was the maximum number of patients that could be examined in order to achieve two groups with an equal median height and interquartile range (median 170 cm, IQR 167–173). Patients with osteoporosis were excluded from the analysis in order to prevent a disproportionate number of shorter men suffering from osteoporosis being matched for height with women. Multivariate, multiple regression analysis showed that gender was not associated with any disease measurement in any of the models tested (Table 9). In contrast, in subgroups of men (n = 50) and women (n = 50) with disparate height, containing males between 176 and 197 cm (median 180 cm), and females between 146 and 156.8 cm (median 153 cm), female sex was associated with a higher DAS28 and HAQ score in models adjusted for other significant variables. No association was seen with MJS (Table S4).

**Table 9:** Multivariate multiple regression analyses showing variables most strongly associated with DAS28, MJS and HAQ score in height-matched male and female patients with RA.

Model 1, dependent variable: DAS28			Model 2, dependent variable: MJS*			Model 3, dependent variable: HAQ		
Independent variable	Regression coefficient (SE)	p value	Independent variable	Regression coefficient (SE)	p value	Independent variable	Regression coefficient (SE)	p value
Comorbid disease†	1.141 (0.395)	0.005	Duration, yrs	0.095 (0.012)	<0.0001	Duration, yrs	0.035 (0.0063)	<0.0001
			CRP, mg/l	0.015 (0.004)	0.001	Carstairs index	0.072 (0.022)	0.002
						ESR, mm/h	0.007 (0.003)	0.006

Variables were baseline values. Based on 54 males with height between 161.4 and 180.5 cm, and 54 females with height between 161.4 and 180.5 cm.

† Presence of any comorbid disease (e.g ischaemic heart disease, diabetes, chronic pulmonary disease, renal disease, neoplasia). Patients with osteoporosis were excluded.

* MJS was square root transformed to fit normality. Gender was not significantly associated in any model.

R-squared values: Model 1 = 0.0741, Model 2 = 0.3347, Model 3 = 0.4066.

### Replication study

Replication of these analyses in a second population of RA patients provided confirmation of the influence of height on disease activity and function. In all patients there was a significant inverse correlation of height with DAS28 (−0.174, p = 0.0030) and HAQ (−0.346, p = 0.0006). The DAS28 and HAQ were again significantly higher in females than males (5.0 v 4.59, p = 0.016 and 1.65 v 1.19, p = 0.0050), but these associations were lost after adjusting for height in multiple regression models (data not shown). The mediation effect also reached significance in this smaller dataset for HAQ (a×b effect: 0.261 (0.074–0.506)).

We also examined subgroups of male and female patients who were either height matched (median 168 cm), or were disparate in height (female median 153 cm, male median 178 cm). Multiple regression analysis on height matched patients showed that gender was not associated with DAS28 (p = 0.20) or HAQ (p = 0.70) in models containing age, disease duration and body weight (or BMI) as independent variables. However, in the subgroup with disparate height, female sex was associated with a higher DAS28 (p = 0.030) and HAQ score (p = 0.0004) in models adjusted for age, disease duration and body weight (or BMI).

## Discussion

This study has demonstrated for the first time that the normal variation of adult height is strongly associated with major disease characteristics in RA. Furthermore, the association of female sex with more active RA and worse function is lost after adjustment for height, suggesting that this relationship may be due to the smaller stature of females. Analysis of the mediation effect showed that height may serve as a full mediator in the relationship of gender with disease activity and overall disability in RA. In gender specific analysis, short stature in male patients was significantly associated with worse disease activity, impairment of mechanical joint function and disability, whilst in females, the relationship was weaker with a significant correlation being found only with disability. However the latter association disappeared when adjusted for other variables associated with the HAQ score.

Different gender specific associations are common in RA, with females often reported as having higher levels of disease activity and worse functional outcome [20]–[25], while males have greater autoantibody production [26]. Higher DAS/DAS28 scores in women have been reported in early disease (<12 months) in some [21], [25], but not all studies [20], [24], but consistently higher female DAS28 scores have been observed in early RA patients followed up prospectively for 5 years [25], and in cross sectional studies of longstanding RA [21], [22]. Higher HAQ scores in women have invariably been found patients with early and established disease [20]–[25]. It has been suggested that higher DAS28 and HAQ in women is possibly due to higher pain perception and less muscular strength. However we did not find a significant difference in pain scores between men and women in this group (unpublished observations). Also, it does not explain why height matched men and women had similar HAQ scores, since we would still have expected the men to have greater muscular strength than the women. Another suggestion is that the menopausal state is responsible for a major part of the differences in outcome between men and women, with postmenopausal women having worse disability and damage [20]. However, we have found that although postmenopausal women have significantly higher DAS28 and HAQ scores than men and premenopausal women, these associations disappear when adjusted for height (unpublished observations).

Further evidence for the influence of height was provided by sub-group analysis which showed no differences in gender for disease activity or functional outcome after matching for height. Differences in height are associated with differences in bone geometry, limb proportions, joint angles, etc. Although height is largely affected by gender, these differences, rather than other sex-related factors (e.g. sex hormones), may be more influential in affecting joint damage.

Body height is likely to be marker for some other environmental or biological process rather than be a causal factor itself. Potential factors influencing height include socio-economic status, smoking, in utero maternal health, nutritional status in childhood and early-life infections. In this study adjustment for the Carstairs deprivation index and smoking history made no difference to the associations with height, but no information was available on childhood infections or nutrition in this cohort. Increased burden of infection, immunity and inflammation in children/teenagers have an adverse impact on linear growth and pubertal development, and hence adult height [8], [12]. Early-onset chronic autoimmune/inflammatory diseases, such as type I diabetes, celiac disease and paediatric inflammatory bowel disease (particularly Crohn's disease), are associated with growth impairment in children/teenagers, although other factors contribute, including nutritional deficit and/or abnormality of the growth hormone/insulin-like growth factor 1 (GH/IGF-1) axis [27]–[29].

Growth hormone (GH) is fundamental to linear growth, and primarily acts as a promoter of skeletal long bone growth/development and chondrocyte maturation [30]. GH stimulates insulin-like growth factor 1 (IGF-1) synthesis, and the latter is responsible for stimulating cartilage and bone extracellular matrix protein synthesis [30]. Deficiency in these proteins thus results in growth retardation, short adult stature, low bone mineral density and bone mass. This would be likely to increase the risk of more severe erosive damage in patients with RA. Recently, a possible association of GH deficiency with autoimmunity and inflammation was suggested [31], [32]. Furthermore, low levels of IGF-1 in RA synovial fluid were found to be associated with systemic inflammation [33]. It is noteworthy that in genome wide association studies (GWAS) a genetic variant in the IFG1 gene was found to be associated with adult height in eastern Asian populations (but not in Caucasians) [34], [35].

It has been proposed that early-life infections may impose a life-long inflammatory burden which may lead to the development of atherosclerotic and thrombotic conditions in late-life [12]. This model also links maternal infections and inflammation with fetal and infant inflammation and reduced growth. In line with this argument, short stature has been associated with increased risk for CVD, type II diabetes and periodontitis [6]–[8]. Interestingly, RA shows numerous characteristics and pathogenetic processes that have similarities to periodontitis [36]. We speculate that this pathophysiological model may provide some explanation for our observation that short stature is associated with more severe RA.

Another important consideration is the effect of the large number of genetic variants which underlie growth/development, and hence adult height. Based on recent large-scale genome-wide association studies (GWAS), a single-nucleotide polymorphism (SNP) in the CDK6 gene, a key controller of cell cycle progression, was found to be associated with both adult height and the susceptibility to RA [37], [38]. GWAS also identified a SNP in the GDF5 gene which is strongly associated both with low adult height and susceptibility to osteoarthritis (OA) [39]–[41]. GDF5 is a member of the TGF-beta superfamily, and is involved in bone growth and differentiation and joint development, both in adult and embryonic tissues [42]–[45]. Rare mutations in the GDF5 gene have been associated with several disorders of skeletal development [39]. The allele, associated with decrease in stature/susceptibility to OA, has been shown to be associated with decreased GDF5 transcriptional activity in chondrogenic cells and with *in vivo* reduced expression of GDF5 in articular cartilage [39], [40]. GDF5 may play a role in repair of damaged joint tissues (e.g. cartilage), and decrease in GDF5 expression may lead to less regeneration.

Other possible genetic candidates include the ESR1 gene which is associated with both adult height and age at onset of RA [2], [46], and several genes implicated in both the extracellular matrix pathway and adult height, including MMP24, ECM2, EFEMP1, ADAMTS13 and ACAN [2], [36], [47]. Further work on the relationship between genetic variants, height and disease severity in RA may provide novel insights into the understanding of the aetiology/pathology of the disease.

There are some limitations to this study due to its cross-sectional nature. Analysis of the relationship between height and disease measures is complicated by secular trends in adult height over recent generations in which there is increased height in younger generations [48]. Furthermore the height in older generations may be reduced due to age related reduction in intervertebral disc height or a loss of vertebral height from osteoporotic vertebral collapse. Adult height also decreases with general ill health and immobility, so the possibility of established, severe RA leading to a greater reduction in height (reverse causation) is a major consideration. However, we found that the inverse association of height with greater disease activity/severity in men remained significant in models adjusted for age, disease duration, osteoporosis, comorbid disease or the presence of other autoimmune conditions. Moreover, other possible confounders such as steroid use, erosive disease, joint replacement, extra-articular disease, presence of autoantibodies and socio-economic status had no significant effect on the relationship between height and disease activity/severity. However, it is clear from the R-squared values in our models that a large amount of the variance in the dependent variables is unexplained by the variables in each of the models. Other unknown/untested factors are therefore important. We were unable to investigate some associations, such as the reported relationship between low serum 25-hydroxyvitamin D (25(OH)D) levels and decreased height [49]. A possible relationship of 25(OH)D deficiency with more severe disease activity in RA [50] suggests that this variable should be considered in future studies on body height and severity of disease.

Further prospective studies of recent onset RA and studies in larger cohorts are needed to confirm our findings. Future studies should also include measurement of leg length since the effects of age and disease severity are likely to have less effect on this component of adult height.

## Supporting Information

Table S1 Multivariate multiple regression analyses showing variables most strongly associated with DAS28, MJS and HAQ score in all patients with RA, excluding adjustment for height.
(DOC)

Table S2 Multivariate multiple regression analyses showing baseline variables most strongly associated with DAS28, MJS and HAQ score over 24 months in all patients with RA.
(DOC)

Table S3 Mediation models demonstrating the mediator effect of height on the relationship between gender and measures of disease activity and outcome over 24 months in all patients with RA.
(DOC)

Table S4 Multivariate multiple regression analysis showing variables most strongly associated with DAS28, MJS and HAQ score in male and female RA patients of disparate height.
(DOC)
