# Supplementary material for: Influence of Adult Height on Rheumatoid Arthritis: Association with Disease Activity, Impairment of Joint Function and Overall Disability
Source: PLoS One. 2013 May 21;8(5):e64862. doi: 10.1371/journal.pone.0064862 (PMC3660323; doi:10.1371/journal.pone.0064862)
Supplement: Table S1 — Multivariate multiple regression analyses showing variables most strongly associated with DAS28, MJS and HAQ score in all patients with RA, excluding adjustment for height. (DOC) [file pone.0064862.s001.doc]

**Table S1.** Multivariate multiple regression analyses showing variables most strongly associated with DAS28, MJS and HAQ score in all patients with RA, excluding adjustment for height.

.

| Model 1, dependent variable: DAS28 | | | Model 2, dependent variable: MJS* | | | Model 3, dependent variable: HAQ | | |
| --- | --- | --- | --- | --- | --- | --- | --- | --- |
| Independent variable | Regression coefficient (SE) | p value | Independent variable | Regression coefficient (SE) | p value | Independent variable | Regression coefficient (SE) | p value |
| RF (+/-) | 0.621 (0.130) | < 0.0001 | Duration, yrs | 0.085 (0.006) | < 0.0001 | Duration, yrs | 0.021 (0.003) | < 0.0001 |
| Comorbid disease^†^ | 0.856 (0.191) | < 0.0001 | Osteoporosis | 0.851 (0.179) | < 0.0001 | ESR, mm/h | 0.009 (0.002) | < 0.0001 |
| Steroid use | 0.502 (0.217) | 0.021 | CRP mg/l | 0.0086 (0.0026) | 0.001 | Carstairs index | 0.062 (0.013) | < 0.0001 |
| Female | 0.280 (0.138) | 0.043 | RF (+/-) | 0.315 (0.126) | 0.013 | Comorbid disease^†^ | 0.323 (0.098) | 0.001 |
|  |  |  |  |  |  | Steroid use | 0.310 (0.115) | 0.007 |
|  |  |  |  |  |  | Female | 0.210 (0.070) | 0.003 |
|  |  |  |  |  |  | Hip replacement | 0.249 (0.118) | 0.036 |

Variables were baseline values. *MJS was square root transformed to fit normality, ^†^Presence of any comorbid disease (e.g ischaemic heart disease, diabetes, chronic pulmonary disease, renal disease, neoplasia). RF, rheumatoid factor; CRP, C-reactive protein; ESR, erythrocyte sedimentation rate. R-squared values: Model 1 = 0.1250, Model 2 = 0.3755, Model 3 = 0.2686.
