# Supplementary material for: Influence of Adult Height on Rheumatoid Arthritis: Association with Disease Activity, Impairment of Joint Function and Overall Disability
Source: PLoS One. 2013 May 21;8(5):e64862. doi: 10.1371/journal.pone.0064862 (PMC3660323; doi:10.1371/journal.pone.0064862)
Supplement: Table S2 — Multivariate multiple regression analyses showing baseline variables most strongly associated with DAS28, MJS and HAQ score over 24 months in all patients with RA. (DOC) [file pone.0064862.s002.doc]

**Table S2.** Multivariate multiple regression analyses showing baseline variables most strongly associated with DAS28, MJS and HAQ score over 24 months in all patients with RA.

.

| Model 1, dependent variable: MTA-DAS28 | | | Model 2, dependent variable: MTA-MJS* | | | Model 3, dependent variable: MTA-HAQ | | |
| --- | --- | --- | --- | --- | --- | --- | --- | --- |
| Independent variable | Regression coefficient (SE) | p value | Independent variable | Regression coefficient (SE) | p value | Independent variable | Regression coefficient (SE) | p value |
| Height | -0.030 (-0.007) | < 0.0001 | Height | -0.017 (0.007) | 0.014 | Height | -0.015 (0.004) | 0.0002 |
| RF (+/-) | 0.478 (0.137) | 0.0006 | Duration, yrs | 0.083 (0.007) | < 0.0001 | Duration, yrs | 0.021 (0.004) | < 0.0001 |
| Comorbid disease^†^ | 0.738 (0.192) | 0.0001 | CRP mg/l | 0.006 (0.003) | 0.023 | Carstairs index | 0.050 (0.014) | 0.0003 |
| Steroid use | 0.502 (0.217) | 0.021 | Steroid use | 0.465 (0.214) | 0.030 | ESR, mm/h | 0.006 (0.002) | 0.0005 |
|  |  |  |  |  |  | Steroid use | 0.298 (0.123) | 0.016 |
|  |  |  |  |  |  | Comorbid disease^†^ | 0.245 (0.106) | 0.021 |
|  |  |  |  |  |  | Hip replacement | 0.297 (0.137) | 0.031 |

MTA: mean-time-averaged values over 24 months, scored at baseline, 12 and 24 months. Based on 305 (72.4%) patients who had been followed up for 24 months with available measurements at each time point. Independent variables were baseline values. *MTA-MJS was square root transformed to fit normality. ^†^Presence of any comorbid disease (e.g ischaemic heart disease, diabetes, chronic pulmonary disease, renal disease, neoplasia). RF, rheumatoid factor; CRP, C-reactive protein; ESR, erythrocyte sedimentation rate. R-squared values: Model 1 = 0.1530, Model 2 = 0.3782, Model 3 = 0.2685.
