# Supplementary material for: Influence of Adult Height on Rheumatoid Arthritis: Association with Disease Activity, Impairment of Joint Function and Overall Disability
Source: PLoS One. 2013 May 21;8(5):e64862. doi: 10.1371/journal.pone.0064862 (PMC3660323; doi:10.1371/journal.pone.0064862)
Supplement: Table S3 — Mediation models demonstrating the mediator effect of height on the relationship between gender and measures of disease activity and outcome over 24 months in all patients with RA. (DOC) [file pone.0064862.s003.doc]

**Table S3.** Mediation models demonstrating the mediator effect of height on the relationship between gender and measures of disease activity and outcome over 24 months in all patients with RA.

| Model 1, dependent variable: MTA-DAS28 | | | | Model 2, dependent variable: MTA-MJS | | | | Model 3, dependent variable: MTA-HAQ | | | |
| --- | --- | --- | --- | --- | --- | --- | --- | --- | --- | --- | --- |
| Path of effect | Regression coefficient (SE) | p value | *Bootstrap results: regression coefficient (95% CI) | Path of effect | Regression coefficient (SE) | p value | *Bootstrap results: regression coefficient (95% CI) | Path of effect | Regression coefficient (SE) | p value | *Bootstrap results: regression coefficient (95% CI) |
| a | -13.761 (0.776) | < 0.0001 | - | a | -13.688 (0.776) | < 0.0001 | - | a | -13.601 (0.772) | < 0.0001 | - |
| b | -0.0310 (0.012) | 0.0074 | - | b | -0.0292 (0.010) | 0.0036 | - | b | -0.0219 (0.006) | 0.0003 | - |
| a×b | 0.427 (-) | - | 0.427 (0.117 – 0.762) | a×b | 0.40 (-) | - | 0.399 (0.135 – 0.689) | a×b | 0.298 (-) | - | 0.296 (0.148 – 0.457) |
| c | 0.375 (0.153) | 0.015 | - | c | 0.148 (0.135) | 0.27 | - | c | 0.199 (0.082) | 0.015 | - |
| c’ | -0.051 (0.219) | 0.82 | - | c’ | -0.253 (0.190) | 0.19 | - | c’ | -0.099 (0.114) | 0.39 | - |

Gender as the independent variable; height as the proposed mediator; disease activity and outcome index (i.e. MTA-DAS28, MTA-MJS or MTA-HAQ) over 24 months as dependent variable. MTA-MJS was square root transformed to fit normality. Models shown adjusted for age and disease duration as standard; adjustment for further confounding factors made no significant difference to the results. Path of effect: a, effect of gender on height; b, effect of height on disease indices; a×b, indirect effect through mediation; c, total effect; c’, direct effect; c can be expressed as the sum of the direct and indirect effects (i.e. c = c’ + a×b). *Bootstrap results for indirect effect through proposed mediation: number of bootstrap resamples, n = 5,000.
