# Supplementary material for: Influence of Adult Height on Rheumatoid Arthritis: Association with Disease Activity, Impairment of Joint Function and Overall Disability
Source: PLoS One. 2013 May 21;8(5):e64862. doi: 10.1371/journal.pone.0064862 (PMC3660323; doi:10.1371/journal.pone.0064862)
Supplement: Table S4 — Multivariate multiple regression analysis showing variables most strongly associated with DAS28, MJS and HAQ score in male and female RA patients of disparate height. (DOC) [file pone.0064862.s004.doc]

**Table S4.** Multivariate multiple regression analysis showing variables most strongly associated with DAS28, MJS and HAQ score in male and female RA patients of disparate height

| Model 1, dependent variable: DAS28 | | | Model 2, dependent variable: MJS* | | | Model 3, dependent variable: HAQ | | |
| --- | --- | --- | --- | --- | --- | --- | --- | --- |
| Independent variable | Regression coefficient (SE) | p value | Independent variable | Regression coefficient (SE) | p value | Independent variable | Regression coefficient (SE) | p value |
| Comorbid disease^†^ | 1.398 (0.375) | 0.0003 | Duration, yrs | 0.068 (0.013) | <0.0001 | Comorbid disease^†^ | 0.732 (0.191) | 0.0002 |
| Female | 0.811 (0.260) | 0.002 | Comorbid disease^†^ | 0.674 (0.352) | 0.059 | Female | 0.384 (0.138) | 0.007 |
|  |  |  | Female | 0.423 (0.245) | 0.088 | Steroid use | 0.535 (0.211) | 0.011 |
|  |  |  |  |  |  | Carstairs | 0.066 (0.025) | 0.013 |

Variables were baseline values. Based on 50 males with height between 176.0 and 197.0 cm, and 50 females with height between 146.0 and 156.8 cm. ^†^Presence of any comorbid disease (e.g ischaemic heart disease, diabetes, chronic pulmonary disease, renal disease, neoplasia). Patients with osteoporosis were excluded. *MJS was square root transformed to fit normality. R-squared values: Model 1 = 0.1996, Model 2 = 0.2605, Model 3 = 0.2989.
